# Editorial: Mechanisms of functional dizziness: a window for understanding space-motion cognition

**DOI:** 10.3389/fneur.2026.1878303

**Published:** 2026-06-03

**Authors:** Hayo Andres Breinbauer, Sofia Waissbluth, Paul Hinckley Delano, Diego Kaski

**Affiliations:** 1 Department of Neuroscience, University of Chile, Santiago, Chile; 2 Department of Otolaryngology, Clínica Alemana de Santiago, Universidad del Desarrollo Facultad de Medicina, Santiago, Chile; 3 Department of Otolaryngology, Pontificia Universidad Catolica de Chile, Santiago, Chile; 4 Servicio de Otorrinolaringologia, Hospital Clinico de la Universidad de Chile Jose Joaquin Aguirre, Santiago, Chile; 5 AC3E, Universidad Tecnica Federico Santa Maria Centro Avanzado de Ingenieria Electrica y Electronica, Valparaíso, Chile; 6 SENSE Research Unit, University College London Institute of Neurology, London, United Kingdom

**Keywords:** chronic dizziness, functional dizziness, perceptual persistent postural dizziness, vestibular cognition, vestibular rehabilitation

Functional dizziness (FD) has emerged as one of the most prevalent chronic vestibular complaints in clinical practice. Persistent postural-perceptual dizziness (PPPD) is its prototypical and most extensively studied entity, but the FD spectrum also encompasses several related conditions, including phobic postural vertigo, visually-induced dizziness, and Mal de Débarquement Syndrome (MdDS). Despite the prevalence and the substantial disability these disorders impose, fundamental questions about their pathophysiology remain unresolved. FD is not characterized by detectable structural damage, nor by primary failure of vestibular afferents and reflex arcs. Instead, the dysfunction appears to reside within the brain's higher-order processing of sensory information—the operations through which multiple inputs are integrated, weighted against internal predictions, and transformed into a coherent representation of the body and the surrounding world. This Research Topic was designed to interrogate that conceptual frontier: what does the brain do with vestibular and visual information beyond reflexive responses, and how do these operations break down in FD?

Framing FD as a disorder of sensory integration and spatial cognition opens a broader scientific window. If the affected processes include the construction of internal maps of the environment, the prediction of self-motion, and the navigation of space, then FD becomes more than a clinical syndrome, it becomes a privileged model to study how the brain assembles space, motion, and self. The four articles gathered in this Research Topic approach this challenge from complementary angles: experimental probes of spatial navigation, computational models of central vestibular dynamics, clinical evaluation of vestibular rehabilitation, and long-term pharmacological outcomes.

Faúndez et al. directly probed the perceptual-cognitive substrate of PPPD using a cross-sectional design with 52 participants: 19 PPPD patients (Bárány Society criteria), 20 vestibular controls with non-PPPD vestibular disorders (benign paroxysmal positional vertigo, vestibular neuritis, vestibular migraine, or Ménière's disease) and quantitatively similar levels of peripheral vestibular dysfunction, assessed by video head impulse test and vestibular evoked myogenic potentials, and 13 healthy volunteers. All participants completed a virtual Morris Water Maze task in both non-immersive (computer-monitor) and immersive virtual reality settings, with concurrent eye-tracking and head-kinematics recording. Beyond classical performance metrics (path length, latency, efficiency, cumulative search error), the authors applied entropy-based measures that quantify the disorganization of navigation trajectories. PPPD patients performed significantly worse than both control groups across all metrics, with disproportionate impairment in tasks demanding allocentric (map-based) navigation. Importantly, performance did not differ between non-immersive and immersive modalities, arguing against visuo-vestibular conflict or sensory overload as the primary driver of the spatial deficit. Increased exploratory gaze behavior in PPPD patients further suggested compensatory strategies for spatial uncertainty. These findings position allocentric mapping and predictive coding errors, rather than sensory input failure, at the core of PPPD-related cognitive dysfunction, and they offer tangible behavioral markers that could inform both diagnosis and therapeutic design.

Complementing this experimental account at the level of central vestibular dynamics, Maruta et al. contribute a model-based exploration focused on MdDS, another subtype of FD. Using a 3 × 3 dynamical-system representation of the velocity storage mechanism, the central vestibular integrator that orients self-motion signals to gravity, the authors examined how attenuating velocity storage, either by shortening its yaw time constant or by reducing its output gain, would affect the alignment between the brain's internal estimate of the vertical and the true gravity vector. Their analysis showed that, when an underlying misalignment is already present (as proposed for the gravitational-pull sensation of MdDS), attenuating velocity storage may paradoxically exaggerate that misalignment rather than correct it. The lesson generalizes beyond MdDS: in functional vestibular disorders, the brain's internal model is the locus of dysfunction, and interventions that reduce sensory contributions without correcting model misalignment can be incomplete or even counterproductive. Their recommendation that targeted optokinetic stimulation may complement habituation protocols echoes, at the level of central vestibular dynamics, the same principle emerging from the experimental work on PPPD: treatment must address how the brain represents space and motion, not only how it suppresses unwanted sensations.

Alahmari and Alshehri evaluated vestibular rehabilitation therapy (VRT) in a cross-sectional comparison between a VRT group (*n* = 94, PPPD diagnosed per Bárány criteria) and a retrospective non-VRT observational cohort (*n* = 94). The VRT protocol combined clinic-supervised sessions twice weekly with daily home exercises over 8 weeks; outcomes were assessed at baseline, post-treatment, and at 12-week follow-up using the EuroQol-5 Dimension (EQ-5D), the Dizziness Handicap Inventory (DHI), and the Hospital Anxiety and Depression Scale (HADS). The authors report better quality of life and lower dizziness-related handicap in the VRT group, while themselves acknowledging that the retrospective, non-randomized design of the comparison cohort invites cautious interpretation. The result we consider most informative is the prognostic weight of anxiety and depression: both emerged as independent predictors of poorer outcome, and longer symptom duration was associated with greater handicap. This finding sharpens a growing recognition that VRT for FD should not be a straightforward transposition of protocols developed for peripheral vestibular deficits. In FD, where the dysfunction lies within the brain's internal model rather than the vestibular periphery, vestibular rehabilitation needs a particular character: one that does not heighten attention to bodily signals or reinforce hypervigilance, but instead encourages the integration of normal vestibular function with attention directed outward, toward the environment rather than the self, expanding metastability to allow the nervous system to update models of the world, while gradually restoring confidence in balance. The rehabilitation of FD, whatever its sensorimotor component, cannot be dissociated from the affective and attentional context in which sensory information is being processed.

Complementing this perspective, Yagi et al. report the first long-term follow-up of serotonergic antidepressants in PPPD diagnosed per Bárány Society criteria. Their retrospective study followed 43 patients (12 men, 31 women; median age 49 years) treated with venlafaxine (*n* = 22), escitalopram (*n* = 10), vortioxetine (*n* = 5), mirtazapine (*n* = 3), or paroxetine (*n* = 3), with assessments at 3 and 6 months and at 1, 1.5, 2, 2.5, and 3 years after initiation. Outcomes were measured with the DHI, HADS, and the Niigata PPPD Questionnaire; a subset of 21 patients also underwent head roll-tilt subjective visual vertical testing as a probe of somatosensory hypersensitivity. Significant improvement was observed across all subjective measures from 3 months onward and was sustained throughout the 3-year follow-up, with an overall adherence rate of 72.9%. Yet a striking observation emerges from the perceptual side of the analysis: head-tilt perception gain, an indicator of somatosensory hypersensitivity in PPPD, did not change after pharmacotherapy, even when subjective symptoms improved. The implication is significant; antidepressants appear to modulate the affective and attentional layers that amplify symptoms, while leaving the underlying perceptual-integrative dysfunction—the layer most directly tied to spatial cognition—relatively untouched.

Read together, these four contributions converge on a coherent thesis. FD is best understood as a discrepancy between the brain's predictive internal model of body, environment, and self-motion and the actual sensory inflow it receives. Symptoms emerge not from broken sensors or reflexes but from miscalibrated internal representations, incorrect assignment of precision of priors, and from the affective and attentional processes that sustain them. Effective management will likely require multimodal strategies that combine pharmacological support for the affective layer, vestibular rehabilitation tailored to the functional context and exploiting exposure rather than reinforcing bodily hypervigilance, and, in a direction this Topic highlights, cognitive interventions targeting spatial mapping and predictive processing. As Topic Editors, we hope this collection contributes to that integrative agenda and helps consolidate FD as a productive model for understanding how the brain builds the space we inhabit.

